# Prevalence of ESBL-Producing *Escherichia coli* on Neck Skin in Slaughtered Broilers Raised on Conventional, Antibiotic-Free, and Organic Farms

**DOI:** 10.3390/pathogens14121265

**Published:** 2025-12-10

**Authors:** Giulia Dilio, Francesca Blasi, Silvia Tofani, Elisa Albini, Serenella Orsini, Marcella Ciullo, Francesca Romana Massacci, Michele Pesciaroli, Giovanni Pezzotti, Chiara Francesca Magistrali

**Affiliations:** 1 Istituto Zooprofilattico Sperimentale Della Lombardia e Dell’Emilia Romagna “Bruno Ubertini”, Via Bianchi 9, 25124 Brescia, Italy; giulia.dilio@izsler.it (G.D.); c.magistrali@izsler.it (C.F.M.); 2 Istituto Zooprofilattico Sperimentale Dell’Umbria e Delle Marche “Togo Rosati”, Via G. Salvemini 1, 06126 Perugia, Italy; francescablasi93@hotmail.it (F.B.); silvia.tofani@izslt.it (S.T.); e.albini@izsum.it (E.A.); s.orsini@izsum.it (S.O.); m.ciullo@izsum.it (M.C.); g.pezzotti@izsum.it (G.P.); 3 Istituto Zooprofilattico Sperimentale del Lazio e Della Toscana “M. Aleandri”, 00178 Rome, Italy; michele.pesciaroli@izslt.it

**Keywords:** *Escherichia coli* ESBL, broiler, slaughter, antimicrobial resistance, farming systems, food safety

## Abstract

Antimicrobial resistance (AMR) among commensal *Escherichia coli* from poultry is a growing concern for food safety and public health. This study investigated AMR patterns in *E. coli* isolated from broiler neck skin at slaughter, comparing organic, antibiotic-free (ATB-free), and conventional production systems. A total of 375 samples were collected from two Italian slaughterhouses and tested by broth microdilution following EU protocols. *E. coli* was recovered from 358 samples, and 37.9% were presumptively positive for ESBL/AmpC-producing strains. Conventional broilers showed the highest resistance to ampicillin (73.8%), sulfonamides (72.5%), and fluoroquinolones (nalidixic acid, 62.5%; ciprofloxacin, 67.5%), while organic and ATB-free systems showed significantly lower levels. Intermediate resistance occurred for trimethoprim (21.4–47.9%) and tetracycline (36–54%), and low prevalence (<10%) was found for gentamicin, tigecycline, and third-generation cephalosporins. No relevant resistance was detected to colistin or carbapenems (≤1.2%). Total *E. coli* counts did not differ among systems, suggesting differences in resistant strain proportions rather than bacterial load. ATB-free flocks processed after conventional batches displayed higher resistance, indicating possible cross-contamination during slaughter. These results highlight the influence of farming practices and slaughterhouse hygiene on AMR dissemination, underscoring the need for integrated farm-to-slaughter control strategies.

## 1. Introduction

Antimicrobial resistance (AMR) has emerged as one of the most pressing global health challenges, affecting both human and veterinary medicine [1,2]. Predictive statistical models suggest that in 2019 bacterial antimicrobial resistance was responsible for approximately 5 million deaths globally [3] and, without the implementation of comprehensive and coordinated interventions, this burden is projected to rise markedly by 2050 [4]. Nevertheless, recent evidence demonstrates that containment strategies—such as national surveillance plans, antimicrobial stewardship programs, and strengthened infection-prevention measures—can contribute to measurable reductions in AMR incidence over time [5,6].

In food-producing animals, antibiotic-resistant *Escherichia coli* act as major carriers of transferable resistance determinants, posing a concern that directly intersects with the One Health perspective [7,8]. According to the Global Burden of Disease analyses, six bacterial species accounted for the majority of AMR-related fatalities in 2019, with *Escherichia coli* emerging as the predominant contributor [3]. Industrial food animal production systems, characterized by high stocking densities and routine antimicrobial administration, create strong selective pressure for resistant strains, facilitating their emergence and dissemination. Within this context, poultry production has been consistently identified as a critical hotspot, underscoring the importance of comparative evaluations between conventional and alternative farming systems to elucidate how distinct production environments shape resistance emergence and dissemination [9,10].

*Escherichia coli* serves as both a ubiquitous commensal and a sentinel for antimicrobial resistance surveillance [11]. In facts, its broad distribution in animal gut microbiota, remarkable ability to acquire mobile resistance elements, and zoonotic relevance via the food chain make it a suitable model for tracking resistance spread [11,12,13]. Particularly worrisome are ESBL-AmpC-producing strains, which often exhibit co-resistance to other antimicrobial classes such as fluoroquinolones, sulfonamides, and tetracyclines [14,15,16]. To better characterize potential dissemination pathways along the poultry production chain, the present study uses neck skin as the sample matrix, a choice that directly reflects contamination occurring at slaughter and thereby enhances the robustness of the comparison between different farming systems. The spread of dominant *E. coli* lineages harboring ESBL determinants, particularly *bla*_CTX-M_ variants, has been reported in both human and veterinary contexts, highlighting how resistance evolves across the entire farm-to-fork chain [9,10]. Recent genomic studies across Europe revealed substantial heterogeneity in ESBL determinants, with clear geographical differences in resistance gene distributions [17].

In recent years, the European Union has strengthened efforts to reduce antimicrobial use in livestock. Legislative measures and consumer-driven demand have contributed to the emergence of alternative production systems, notably organic and antibiotic-free (ATB-free) broiler farming [18]. These systems are characterized by the absence of or a drastic reduction in antimicrobial treatments, lower stocking densities, and enhanced biosecurity measures [19]. Evidence from different studies indicates that broilers raised under alternative or non-conventional production systems tend to show a lower prevalence of resistant *E. coli* in their caecal contents compared with broilers from intensive systems [20,21,22]. Differences in loads and in the profiles of virulence and resistance genes among *E. coli* isolates from diverse production contexts further support the hypothesis that management practices and antimicrobial exposure can influence the selection pressure and overall resistance levels. Recent global reviews support this evidence, showing that organic and antibiotic-free farms generally exhibit lower levels of AMR compared with conventional systems, although important variations exist between countries and farming conditions [23].

However, the relationship between farming systems and AMR cannot be entirely explained by antimicrobial use alone. Environmental factors, biosecurity practices, vertical transmission from breeders and the slaughtering process may contribute to shaping the final contamination profile observed on poultry carcasses [11,24,25,26]. Slaughterhouses represent a key node in this chain: previous studies have shown that resistant strains can persist and spread within the slaughterhouse environment, raising concerns that the benefits of antimicrobial reduction at the farm level may be partially offset by cross-contamination during processing [21]. This has been further confirmed by recent Italian studies, which revealed high genomic diversity and persistence of ESBL *E. coli* in slaughterhouse environments, even in regions with reduced antimicrobial use at the farm level [27].

The choice of sample matrix is also critical for assessing AMR exposure risk. While caecal content has been widely used as a proxy for intestinal carriage, it does not directly reflect what consumers are exposed to. In contrast, neck skin samples are recognized by EFSA as a reliable indicator of carcass hygiene and microbial contamination at slaughter [24,28]. Monitoring resistance in neck skin isolates is therefore highly relevant for food safety, as it provides a direct measure of potential consumer exposure to resistant bacteria [29]. Despite this, most research to date has focused on caecal content or retail meat [30,31], with limited studies addressing neck skin contamination in relation to farming systems. Recent work in Europe and Asia (Malaysia) has shown that ESBL-producing *E. coli* is also widespread in farm environments and broilers, underscoring the importance of considering both pre-harvest and slaughterhouse contamination routes [32,33].

A further layer of complexity is introduced by the molecular epidemiology of resistant *E. coli*. Genes encoding ESBLs, such as *bla*_CTX-M_ (groups 1, 2, 9, 8/25), *bla*_TEM_, and *bla*_SHV_, as well as plasmid-mediated AmpC genes (*bla*_CMY_, *bla*_CIT_, *bla*_DHA_, *bla*_FOX_, etc.), are widely distributed across both human and animal isolates [27,32,34]. Recent systematic reviews confirm that the global prevalence of ESBL-producing *E. coli* in farm animals has risen sharply in the last five years, reflecting the spread of mobile resistance determinants across species and geographical regions [35].

These aspects highlight the importance of assessing AMR not only at farm level but also at slaughter, where consumer exposure is most direct. Yet there remains a lack of data on neck skin contamination of broilers across different production systems. Understanding whether the lower prevalence of resistance observed in caecal contents of organic and ATB-free broilers translates into a tangible reduction in resistant *E. coli* on carcasses is essential for evaluating the true public health benefits of these systems [36,37,38].

The present study addresses this gap by investigating the prevalence and resistance profiles of *E. coli* isolated from neck skin of broilers raised in conventional, organic, and ATB-free systems. Furthermore, to explicitly assess the role of slaughterhouse practices in resistance dissemination, the study evaluates the impact of the slaughter sequence on cross-contamination, with particular focus on ATB-free flocks processed before or after conventional batches. By integrating phenotypic resistance data with molecular characterization of ESBL/AmpC genes, this study contributes to a more comprehensive understanding of how farming practices and slaughterhouse dynamics shape AMR at the consumer interface.

## 2. Materials and Methods

### 2.1. Study Design

The sampling strategy was designed to evaluate potential differences in antimicrobial resistance profiles of *E. coli* isolates between production systems and slaughterhouses. The specific objectives were:

▪ To verify whether the resistance levels detected in caecal contents were also observed on carcasses (neck skin);
▪ To assess potential differences between the two slaughterhouses for antibiotic-free (ATB-free) flocks;
▪ To investigate whether slaughtering ATB-free flocks after conventional ones could increase the risk of resistance contamination and, based on a priori hypothesis, to determine whether Group C (ATB-free slaughtered after conventional flocks) would show a higher prevalence of resistant *E. coli* compared with Group D (ATB-free slaughtered before conventional flocks).

Samples originated from a large integrated poultry company which employs three production systems: conventional, organic, and antibiotic-free (ATB-free).

In slaughterhouse 1, organic (group A) and ATB-free (group B) broilers were slaughtered on separate days. In slaughterhouse 2, both conventional (group E) and ATB-free broilers were slaughtered on the same day. For this slaughterhouse, two ATB-free groups were considered depending on slaughter sequence: ATB-free after conventional (group C) and ATB-free before conventional (group D).

The sample size was calculated assuming a 50% expected prevalence of resistant *E. coli*, 15% precision and 95% confidence level, resulting in a minimum of 285 samples distributed into the five categories listed below. The plan was subsequently adjusted according to field feasibility and slaughterhouse availability, leading to a final sample size greater than initially estimated, thus improving representativeness (Table 1). Neck skin samples were collected at the end of processing from two poultry slaughterhouses, immediately stored at +4 °C, and analyzed within 12 h. Both slaughterhouses operated under comparable hygiene standards and routine line speeds during all sampling days; although minor day-to-day operational variability cannot be entirely excluded, no deviations from standard processing conditions were reported, thereby minimizing the influence of unmeasured confounders on the sequence-based comparison.

### 2.2. Sampling

Between February and May 2019, 375 neck skin samples were collected from broiler chickens slaughtered at two Italian poultry slaughterhouses (slaughterhouse 1 and slaughterhouse 2). For each category, the sampling plan was defined using an assumed prevalence of 50%, a 95% confidence level, and a precision of 15%. The plan was subsequently adjusted according to field feasibility and slaughterhouse availability, leading to a final sample size greater than initially estimated, thus improving representativeness (Table 1).

### 2.3. Microbiological Analyses

#### 2.3.1. Bacterial Enumeration

Quantitative culture was used to determine the neck skin loads of *E. coli* and *E. coli* resistant to 3rd generation cephalosporin and to nalidixic acid. Briefly, approximately 25 g of neck skin were homogenized in buffered peptone water into a Stomacher bag. The suspension (10^−1^ dilution *w*/*v*) was further 10-fold diluted in 0.9% saline. The colony-forming units (CFU) of *E. coli* of interests were determined by plating 100 μL of each dilution on MacConkey agar for total *E. coli* count, MacConkey agar supplemented with cefotaxime (1 µg/mL) (Sigma Aldrich—Merck KGaA, Darmstadt, Germany) for the isolation of 3rd generation cephalosporin-resistant *E. coli*, MacConkey agar supplemented with nalidixic acid (50 µg/mL) (Sigma Aldrich—Merck KGaA, Darmstadt, Germany) for the isolation of quinolone-resistant *E. coli*.

Colony counts were expressed as log_10_ CFU/g of skin. For negative results, 10 µL of buffered peptone water incubated at 37 °C for 24 h was plated and only presence/absence recorded. One colony per plate was sub-cultured on TSA and confirmed by MALDI-TOF MS (Bruker Daltonics, Bremen, Germany).

#### 2.3.2. Antimicrobial Susceptibility Testing

One typical colony per sample was selected at random and *E. coli* isolates from MacConkey agar (n = 358) were tested for antimicrobial susceptibility by broth microdilution using Sensititre EU Surveillance *Salmonella*/*E. coli* EUVSEC plates (Thermo Fisher Scientific, Waltham, MA, USA), in accordance with Commission Decision 2013/652/EU [39]. The EUVSEC panel (currently commercialized as EUVSEC3) included the following antimicrobials and concentration ranges (mg/L): ampicillin (AMP, 1–64), azithromycin (AZI, 2–64), cefotaxime (FOT, 0.25–4), ceftazidime (TAZ, 0.5–8), chloramphenicol (CHL, 8–128), ciprofloxacin (CIP, 0.015–8), colistin (COL, 1–16), gentamicin (GEN, 0.5–32), meropenem (MEM, 0.03–16), nalidixic acid (NAL, 4–128), sulfamethoxazole (SMX, 8–1024), tetracycline (TET, 2–64), tigecycline (TGC, 0.25–8), and trimethoprim (TMP, 0.25–32).

A total of 142 *E. coli* isolates recovered on MacConkey agar supplemented with cefotaxime was further tested for ESBL/AmpC phenotypes using Sensititre EU Surveillance ESBL/AmpC EUVSEC2 plates (Thermo Fisher Scientific). The EUVSEC2 panel included: cefepime (FEP, 0.06–32), cefotaxime (FOT, 0.25–64), cefotaxime/clavulanic acid (F/Cl, 0.06/4–64/4), cefoxitin (FOX, 0.5–64), ceftazidime (TAZ, 0.25–128), ceftazidime/clavulanic acid (T/Cl, 0.12/4–128/4), ertapenem (ETP, 0.015–2), imipenem (IMI, 0.12–16), meropenem (MEM, 0.03–16), and temocillin (TRM, 0.5–128). For wells containing clavulanate, the inhibitor was present at a fixed concentration (4 mg/L). *E. coli* strain ATCC 25922 served as the quality-control strains.

Minimum Inhibitory Concentration (MIC) determination was performed on all *E. coli* isolates recovered from both unsupplemented (EUVSEC panel, n = 358) and cefotaxime-supplemented (EUVSEC 2 panel, n = 142) MacConkey agar using the broth microdilution method. While MIC results from EUVSEC isolates were used to estimate antimicrobial resistance prevalence, EUVSEC 2 tested isolates served for the qualitative assessment of presumptive ESBL/AmpC resistance, later confirmed by PCR.

MIC values were interpreted according to the epidemiological cutoff values (ECOFFs) defined by EFSA in 2024 [40], and percentages of non-wild-type (NWT) isolates are reported as resistance rates.

### 2.4. Molecular Characterization

The 142 isolates collected from MacConkey agar supplemented with cefotaxime (1 µg/mL) and tested for antimicrobials with EUVSEC2 panels were subjected also to molecular analysis for the detection of ESBL/AmpC genes. Based on the phenotypic interpretation, isolates were preliminarily categorized as presumptive AmpC or ESBL-producing. Subsequent molecular analysis was therefore targeted to the corresponding resistance genes.

Genomic DNA was extracted using the QIAamp DNA Mini Kit (Qiagen, Hombrechtikon, Switzerland), according to the manufacturer’s instructions.

The detection of ESBL and AmpC-associated genes was carried out following the protocol described by Tofani et al. [21] with additional methodological details as follows.

Briefly, isolates recovered on MacConkey agar supplemented with cefotaxime were screened by PCR for the following targets:

- ESBL genes: *bla*_TEM_, *bla*_SHV_, *bla*_OXA_ (multiplex PCR); *bla*_CTX-M_ groups 1, 2, and 9 (multiplex PCR) [41];
- Isolates positive for *bla*_CTX-M_ group 1 were further analyzed by a specific simplex PCR for the allelic variant *bla*_CTX-M-15_ [42];
- AmpC genes: *bla*_ACC_, *bla*_FOX_, *bla*_MOX_, *bla*_DHA_, *bla*_CIT_, and *bla*_EBC_ (multiplex PCR) [43];
- Isolates positive for *bla*_CIT_ were tested by a simplex PCR for the detection of *bla*_CMY-2_ [44].

The PCR protocol was largely consistent across target genes, with minor target-specific modifications reported in the cited references [21,41,42,43,44]. Each 25 µL reaction contained approximately 50 ng of template DNA, 1 U of Taq DNA polymerase, 2.5 µL of 10× buffer, 1.5 mM MgCl_2_, 0.2 mM dNTPs, and primers at 0.4–0.5 µM. Thermal cycling included an initial denaturation at 94–95 °C for 5 min, followed by 30–35 cycles of denaturation at 94–95 °C for 30 s, annealing at 58–60 °C for 30 s, and extension at 72 °C for 1 min, with a final extension at 72 °C for 5–7 min. PCR products were analyzed by agarose gel electrophoresis (1.5% agarose, TBE buffer, Promega, Milan, Italy). Reference strains carrying known ESBL and AmpC genes were included as positive controls.

### 2.5. Statistical Analysis

The bacterial counts were converted to log_10_ CFU/g of neck skin for statistical analysis and tested for normality (Shapiro–Wilk test). Since distributions were non-normal, Kruskal–Wallis tests were applied to compare groups, followed by pairwise Wilcoxon rank-sum tests with Bonferroni correction.

Prevalence data were summarized as percentages with corresponding 95% confidence intervals (CIs). Differences in prevalence between production systems were assessed using Chi-square or Fisher’s exact tests, as appropriate. Pearson’s Chi-square test was used when all expected cell counts were ≥5, whereas Fisher’s exact test was applied in cases where one or more expected frequencies fell below this threshold, in accordance with standard statistical recommendations. In addition, odds ratios (*ORs*) with 95% CIs were calculated for each group with the conventional production type as a reference (*OR* = 1). A *p*-value < 0.05 was considered statistically significant. All analyses were performed in R version 4.5.0 (R Core Team, 2025) within RStudio version 2025.05.1+513 (Posit Software, PBC, Boston, MA, USA).

## 3. Results

Out of the 375 samples, *E. coli* was successfully isolated from 358 (95.5%), which were included in the subsequent analyses, while 17 samples were negative for *E. coli* growth.

### 3.1. Enumeration of E. coli: Quantitative Analysis

**Figure 1:**
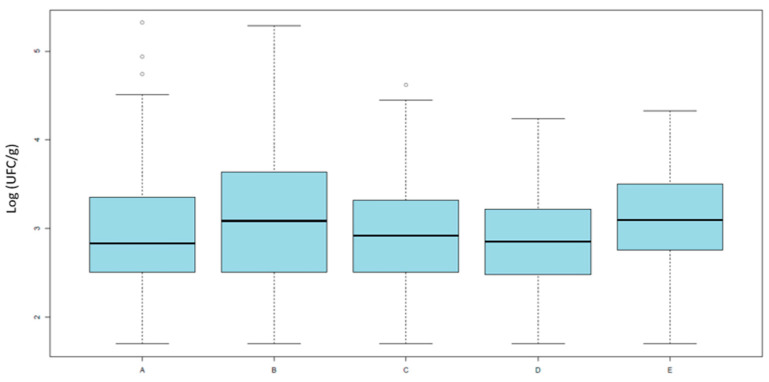
Results of quantitative analysis. Averages of *E. coli* loads (Log_10_ UFC/g) isolated on MacConkey agar across the five categories. Category A includes organic broilers processed in slaughterhouse 1; Category B includes antibiotic-free broilers also from slaughterhouse 1; Categories C and D include antibiotic-free broilers processed in slaughterhouse 2, respectively, after or before conventional batches; and Category E includes conventional broilers processed in slaughterhouse 2.

The data did not follow a normal distribution (Shapiro–Wilk test, *p* < 0.0001), the distribution of total *E. coli* counts on MacConkey agar (Figure 1) did not significantly differ between groups (Kruskal–Wallis *χ*^2^ = 8.58, *df* = 4, *p* = 0.0724), indicating that the overall level of *E. coli* contamination was comparable across groups.

This suggests that the observed differences in antimicrobial resistance are not attributable to variations in bacterial load, but rather to the relative proportion of resistant strains within the *E. coli* population.

**Figure 2:**
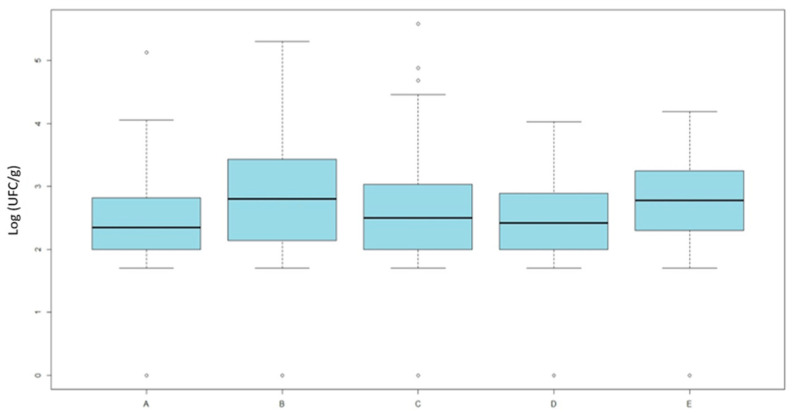
Results of quantitative analysis. Averages of *E. coli* loads (Log UFC/g) isolated on MacConkey agar supplemented with nalidixic-acid across the five categories. Category A includes organic broilers processed in slaughterhouse 1; Category B includes antibiotic-free broilers also from slaughterhouse 1; Categories C and D include antibiotic-free broilers processed in slaughterhouse 2, respectively, after or before conventional batches; and Category E includes conventional broilers processed in slaughterhouse 2.

Counts on MacConkey agar supplemented with nalidixic acid showed significant differences (Kruskal–Wallis, *χ*^2^ = 17.98, *df* = 4, *p* = 0.0012) (Figure 2).

Pairwise comparisons were performed using the Wilcoxon rank-sum test with Bonferroni adjustment. Post hoc Wilcoxon tests revealed that organic broilers (group A) had significantly lower counts of quinolone-resistant *E. coli* compared with ATB-free broilers (group B, *p* = 0.049) and conventional broilers (group E, *p* = 0.006). Additionally, ATB-free broilers slaughtered before conventional (group D) differed from the conventional group (*p* = 0.048), while no difference was observed for those slaughtered after (group C).

However, the difference between groups C and D was minimal, as confirmed by the graphical distribution and the non-significant *p*-value reported in Table 2.

On MacConkey agar supplemented with cefotaxime, counts were low and frequently below the detection limit.

The Kruskal–Wallis test indicated significant differences among groups (*χ*^2^ = 12.57, *df* = 4, *p* = 0.0136). Moreover, most observations fell below the detection threshold or were only positive after pre-enrichment, making quantitative analysis less informative. For this reason, results were transformed into a qualitative outcome (positive/negative growth). A sample was considered ESBL-positive if any growth was detected on MacConkey agar supplemented with cefotaxime. The count of ESBL-positive *E. coli* also included presumptive AmpC producers.

The distribution of ESBL positive samples was as follows: 33.3% in group A (25/75), 26.2% in group B (17/65), 42.9% in group C (45/105), 30% in group D (15/50), and 50.0% in group E (40/80), corresponding to an overall prevalence of 37.9% (142/375) (Table 3).

Results of the odds ratio for ESBL/AmpC positivity *E. coli*, using the conventional production type as a reference (group E), are summarized in Table 4.

Given the relevance of slaughter sequence in shaping potential cross-contamination dynamics, the following results specifically address the comparison between ATB-free flocks processed before or after conventional batches. This aspect is particularly noteworthy, as the observed patterns partially diverge from expected contamination trends.

These results describe a protective effect of ATB-free and organic farming against ESBL-AmpC *E. coli* contamination. This effect was significant for group B (ATB-free broilers from slaughterhouse 1) and group D (ATB-free slaughtered before conventional batches at slaughterhouse 2). Protective but borderline effect is significant (*OR* = 0.50, 95% CI = 0.24–1.00, *p* = 0.049) in group A (organic production system). No significant protection was observed when ATB-free broilers were slaughtered after conventional flocks (group C). Nonetheless, the contrast between groups C and D highlights the potential impact of slaughter sequence on contamination risk, although the difference between groups C and D was modest, as reflected by the prevalence values and the overlapping 95% confidence intervals.

### 3.2. Antimicrobial Susceptibility Profiles

Both *E. coli* isolates obtained from MacConkey agar (n = 358) and those recovered from MacConkey agar supplemented with cefotaxime (n = 142) were tested by MIC. The two groups were analyzed for different purposes. Total *E. coli* isolates from MacConkey, representative of commensal *E. coli*, were used to assess antimicrobial resistance prevalence across farming systems. Conversely, *E. coli* isolates from MacConkey supplemented with cefotaxime, presumptively selected for resistance to third-generation cephalosporins, were evaluated qualitatively to estimate the occurrence of ESBL and/or AmpC-producing *E. coli*. MIC data obtained using EUVSEC 2 panel were not included in prevalence calculations but used to support the presumptive classification subsequently confirmed by molecular testing.

**Figure 3:**
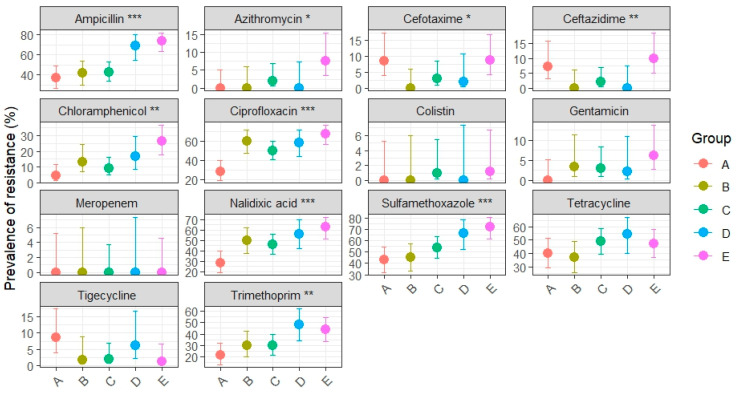
Dot-and-whisker plot of resistance prevalence (%) with 95% CIs for total *E. coli* from MacConkey agar isolated from broiler neck skin, stratified by category (A: organic, B: antibiotic-free, C: ATB-free after conventional, D: ATB-free before conventional, E: conventional). Asterisks indicate the level of statistical significance for overall group differences (Chi-square or Fisher’s exact test): *p* < 0.05 (*), *p* < 0.01 (**), *p* < 0.001 (***).

Phenotypic resistance patterns of *E. coli* isolates from MacConkey agar and tested with EUVSEC panel are summarized in Appendix A, Table A1, while the distribution of MIC values for each antimicrobial agent is presented in Supplementary Table S1. Figure 3 illustrates prevalence estimates with 95% CIs across production systems with statistical significance for overall group differences (Chi-square or Fisher’s exact test).

Ampicillin resistance was not evenly distributed across production systems (*p* < 0.001), with the highest prevalence observed in conventional broilers (73.8%, 95% CI: 63.2–82.1), significantly exceeding that in organic (37.1%, 95% CI: 26.8–48.9), ATB-free (43.3%, 95% CI: 30.1–54.3), and ATB-free broilers slaughtered after conventional groups (42.6%, 95% CI: 33.7–52.8). ATB-free broilers slaughtered before conventional batches (group D) showed very high resistance (68.8%, 95% CI: 54.7–80.1), closer to conventional broilers, likely reflecting cross-contamination during slaughter processing.

Ciprofloxacin resistance also differed significantly (*p* < 0.001) among groups, with conventional broilers showing the highest prevalence (67.5%, 95% CI: 56.0–77.2), significantly greater than organic (28.6%, 95% CI: 19.3–40.1) and ATB-free broilers slaughtered after conventional groups (50.0%, 95% CI: 37.4–62.6). Both ATB-free groups from slaughterhouse 1 and slaughterhouse 2 (before conventional) showed unexpectedly very high prevalence, of 60% (95% CI: 47.4–71.4) and 58.3% (95% CI: 44.3–71.2), respectively.

Similar patterns were observed for nalidixic acid (*p* < 0.001) and chloramphenicol (*p* < 0.01). Nalidixic acid resistance was markedly higher in conventional broilers (62.5%, 95% CI: 51.5–72.3) than organic (28.6%, 95% CI: 19.3–40.1); whereas chloramphenicol showed an increasing gradient from organic (4.3%, 95% CI: 1.5–11.9) to conventional (26.2%, 95% CI: 17.9–36.8). Ciprofloxacin, nalidixic acid and chloramphenicol intermediate ATB-free groups displayed overlapping CIs.

Resistance to sulfamethoxazole showed consistently high prevalence across all production systems (*p* < 0.001). The highest rate was in conventional broilers (72.5%, 95% CI: 61.9–81.1), followed by D group (66.7%, 95% CI: 52.5–78.3) but with substantial levels also in ATB-free groups A-B (from 45% to 54%, overlapped CIs) and organic group (42.9%, 95% CI: 31.9–54.5).

Trimethoprim demonstrated differences among production systems (*p* < 0.01), with groups D and E clustering at higher prevalence values compared to other groups (D: 47.9%, 95% CI: 34.5–61.7; E: 43.8%, 95% CI: 33.4–54.7).

Differences were also observed for ceftazidime (*p* < 0.01), azithromycin and cefotaxime (*p* < 0.05). Although all rates fell within the EFSA “low resistance” range (1–10%) [45], ceftazidime and cefotaxime resistance were relatively higher in the conventional group (CEZ: 10%, CEF: 8.8%) compared to the organic group (CEZ: 7.1%, CEF: 8.6%). Conversely, tetracycline resistance was uniformly high across all groups (36–54%), with overlapping confidence intervals indicating no significant differences.

For gentamicin, tigecycline, cefotaxime and ceftazidime prevalence were low (0–10%) and confidence intervals were wide and largely overlapping, suggesting limited epidemiological relevance. No resistance was observed to meropenem or colistin.

These findings demonstrate that antimicrobial resistance is not homogeneously distributed across production systems, with conventional broilers consistently showing the highest prevalence. Organic and ATB-free systems generally displayed reduced resistance, except where cross-contamination during slaughter likely influenced outcomes.

### 3.3. Molecular Characterization

Prior to molecular analysis, all isolates obtained from MacConkey agar supplemented with cefotaxime were tested for MIC values and, based on the phenotypic interpretation, they were preliminarily categorized as presumptive AmpC or ESBL-producing strains. Subsequent PCR screening was therefore targeted to the corresponding resistance genes.

A total of 142 isolates were analyzed. Based on phenotypic interpretation, 129/142 (90.8%) isolates were initially categorized as presumptive ESBL producers and 12/142 (8.5%) as presumptive AmpC producers. Among the presumptive ESBL producers, 128/129 (99.2%) were confirmed to carry ESBL genes, while among the presumptive AmpC producers, 11/12 (91.7%) were confirmed to carry AmpC genes.

Overall, molecular testing revealed that 128/142 (90.2%) were positive for ESBL genes and 11/142 (7.7%) were positive for AmpC genes. Only 3/142 (2.1%) isolates that were initially classified as presumptive positive were negative for all tested genes. Distributions of detected genes are described in Table 5.

Statistical analyses of ESBL and AmpC gene prevalence across production systems were performed using Fisher’s Exact Test. No significant differences were observed for either ESBL (*p* = 0.869) or AmpC (*p* = 0.617) gene distribution among the groups, indicating that the occurrence of these resistance genes was similar across the different production systems.

## 4. Discussion

The present study demonstrated that poultry production systems strongly influence the prevalence of antimicrobial-resistant *Escherichia coli* on broiler neck skin and consequently the carcasses.

The quantitative results indicate that the overall commensal *E. coli* load on neck skin samples did not significantly differ among production systems, suggesting that the total level of bacterial contamination was comparable across groups. This observation implies that differences in antimicrobial resistance are not attributable to variations in bacterial load, but rather to the relative abundance of resistant strains within the *E. coli* population. Similar findings have been reported in previous studies showing that total commensal *E. coli* counts on poultry carcasses are generally independent of farming type [29,46]. However, when focusing on quinolone- and cephalosporin-resistant *E. coli*, significant differences emerged between production categories. Organic and antibiotic-free (ATB-free) broilers showed lower counts of resistant *E. coli* compared with conventional ones, particularly when ATB-free flocks were processed before conventional batches. Conversely, ATB-free broilers slaughtered after conventional flocks exhibited resistance levels comparable to conventional systems, indicating a potential carry-over effect due to cross-contamination during processing. This pattern aligns with previous studies demonstrating that slaughter sequence and equipment hygiene play a crucial role in the dissemination of resistant bacteria, with surfaces and tools acting as reservoirs facilitating transfer between flocks [47,48]. Additionally, these findings extend previous observations on caecal contents [20,30,31] to the slaughterhouse stage, where neck skin represents a relevant indicator for carcass contamination [24]. Our results generally align with previous studies conducted on caecal contents [20,21,22], which, although not directly comparing caecal and carcass isolates from the same animals, supporting the view that the intestinal microbiota represents the primary source of contamination during slaughter. Resistance prevalence on carcasses tends to be slightly lower—mainly due to processing steps such as scalding, defeathering, and washing—while overall resistance profiles remain comparable.

Conventional broilers harbored the highest prevalence of resistant *E. coli*, particularly to ampicillin (73.8%) and sulfamethoxazole (72.5%), with confidence intervals clearly separating this group from organic (ampicillin 37.1%, sulfamethoxazole 42.9%) and ATB-free broilers (41.7–54%). Resistance to fluoroquinolones (ciprofloxacin, nalidixic acid) followed a similar pattern, with significantly higher levels (62.5–67.5%) in conventional compared to organic broilers (28.6% for both). Ampicillin, sulfonamides, and fluoroquinolones remain key markers of resistance in poultry *E. coli* [14,15,16,47]. These trends are consistent with the pan-European survey conducted in 2022 [49], which reported variable but generally high resistance levels in commensal *E. coli* from broilers, particularly to ampicillin and sulfonamides. However, the levels observed in our study were often higher, possibly reflecting local differences in antimicrobial use. Differences in production system likely reflect the overuse of antimicrobial and higher stocking densities in conventional systems [30,50], while organic and ATB-free standards reduce selective pressure for resistant strains [51]. Importantly, no resistance was observed to carbapenems (meropenem) or colistin, drugs of last resort in human medicine, confirming generally EU-wide surveillance trends in 2025 [7]. Although resistance to these last-resort antibiotics was generally absent across the EU, isolated reports documented *E. coli* isolates from healthy broilers carrying the plasmid-borne *mcr*-1 and *bla*_NDM_ genes [52], underscoring the One Health risk posed by antimicrobial resistance in poultry. Resistance to cefotaxime and ceftazidime in *E. coli*, proxies for ESBL production, was detected at low but significant levels, with the highest prevalence in conventional raised broilers. The notably lower resistance rates found in organic and ATB-free production systems further reinforce the hypothesis that restricting antimicrobial use in livestock can effectively reduce the selection and dissemination of resistant bacterial strains.

Although phenotypic resistance varied among production systems, molecular analysis showed no significant differences in the distribution of ESBL/AmpC genetic determinants. This apparent discrepancy is well described in the literature: the presence of a resistance gene does not necessarily result in its phenotypic expression, as transcriptional regulation, promoter variation, gene copy number or mutations can modulate expression levels [53,54,55]. Moreover, additional mechanisms—such as efflux pump overexpression or porin alterations—may contribute to resistance independently of β-lactamase genes [53,56]. Finally, molecular assays detect the presence of genes within the population, whereas phenotypic tests reflect the fraction of cells actively expressing resistance at the time of testing. The expression of resistance genes is also strongly influenced by environmental conditions—including antimicrobial exposure, microbial interactions, disinfectants and other environmental stressors—which may differ across production systems even when the same genetic determinants are widely disseminated [21,53].

This interpretation is consistent with reports from other European studies [32,34], which also observed widespread dissemination of *bla*_CTX-M_ and *bla*_CMY_ genes regardless of production type. Our findings do not always fully align with a previous study [21], which observed a reduced prevalence of resistance in organic and ATB-free poultry systems. However, this divergence may be partly attributable to methodological differences and to the type of matrix analyzed (caecal content versus neck skin in our study), primarily reflecting the intestinal microbiota and internal colonization patterns.

The slaughter sequence played a critical role in shaping resistance prevalence: this observation aligns with previous reports highlighting the impact of slaughterhouse hygiene practices and sequencing on cross-contamination [57,58]. The results observed for Groups C and D were partially counter-intuitive, as ATB-free broilers processed before conventional batches unexpectedly showed resistance levels close to conventional broilers, whereas ATB-free flocks slaughtered after conventional displayed lower rates. This apparently contradicts the biological expectation that flocks processed after conventional broilers would be more contaminated and diverges from the expected pattern and from previous evidence [20,21], suggesting that slaughterhouse environment or residual contamination may play a larger role. This complexity should be interpreted with caution, as the sample size available for the sequence sub-study may have limited the statistical power to detect clearer differences. Moreover, unmeasured confounding factors—such as day-specific variations in water quality, ambient temperature, or operational conditions unique to Slaughterhouse 2—may also have influenced cross-contamination dynamics. To address this issue in more detail, several hypotheses can be considered.

First, groups C–E were processed in slaughterhouse 2, where conventional broilers were routinely slaughtered, possibly leading to a baseline environmental contamination that already affected group D. In contrast, slaughterhouse 1 (processing only organic and ATB-free) consistently showed lower resistance levels. Second, hygiene procedures such as partial cleaning and disinfection between production batches could have reduced residual contamination, mitigating the expected increase in group C. Finally, group D included only 50 samples compared with 105 in group C, reducing statistical power and resulting in wider confidence intervals, which weakens the robustness of the comparisons.

In addition, our finding of unexpectedly high ciprofloxacin resistance in *E. coli* isolates from ATB-free broiler, particularly those slaughtered before conventional batches, is consistent with previous reports documenting substantial fluoroquinolone resistance even in antibiotic-free production systems [16].

## 5. Conclusions

This study demonstrated that both production systems and slaughter practices significantly influence the prevalence of antimicrobial-resistant *E. coli* on broiler neck skin, with potential implications for bacterial contamination of carcasses. Broilers raised under organic and antibiotic-free conditions generally exhibited lower resistance levels compared to those from conventional systems, supporting the protective effect of reduced antimicrobial use. Molecular analysis further showed that 128 of 142 isolates (90.2%) carried ESBL genes and 11 of 142 (7.7%) harbored AmpC genes, whereas only 3 of 142 isolates (2.1%) initially classified as presumptive positives tested negative for all targeted genetic determinants.

The role of production systems and slaughterhouse practices in shaping antimicrobial resistance highlights the need for further investigation. Future studies could integrate genomic approaches to better characterize circulating *E. coli* strains across different production systems, providing deeper insights into AMR dynamics along the food chain and supporting the development of effective mitigation strategies.

These results highlight that not only farm management but also the slaughterhouse environment and hygiene strongly influence AMR spread. Improving slaughter hygiene practices and expanding non-conventional production systems may reduce the dissemination of resistant bacteria along the food chain, thereby contributing to a lower public health risk associated with resistant *E. coli* and reinforcing the One Health perspective.

## Figures and Tables

**Table 1:** Number of neck skin samples collected from broilers raised in conventional, antibiotic-free, and organic production systems across the different categories and slaughterhouses.

Categories	Slaughterhouse	Production System	Number of Samples
A	1	Organic	75
B	1	ATB-free	65
C	2	ATB-free (AFTER conventional)	105
D	2	ATB-free (BEFORE conventional)	50
E	2	Conventional	80
Total of samples			375

**Table 2:** Pairwise comparison between different broiler production systems (Wilcoxon rank sum test with Bonferroni correction). Reported values are *p*-values; dashes indicate redundant comparisons (already shown in the symmetric cell).

Category	A	B	C	D	E
**A**	**-**	0.049 *	1	1	0.006 **
**B**	0.049 *	-	0.511	0.197	1
**C**	1	0.511	-	1	0.137
**D**	1	0.197	1	-	0.048 *
**E**	0.006 **	1	0.137	0.048 *	-

**Table 3:** Prevalence of presumptive ESBL/AmpC positive *E. coli* in neck skin samples isolated from MacConkey supplemented with cefotaxime, divided by category, with 95% confidence intervals (CIs 95%).

Category	ESBL-AmpC-Negative	ESBL-AmpC-Positive	CI 95%	Total
A	50 (66.7%)	25 (33.3%)	(23.7–44.6)	75
B	48 (73.8%)	17 (26.2%)	(17.0–38.0)	65
C	60 (57.1%)	45 (42.9%)	(33.8–52.4)	105
D	35 (70%)	15 (30%)	(19.1–43.8)	50
E	40 (50%)	40 (50%)	(39.3–60.7)	80
Total	233 (62.1%)	142 (37.9%)	(33.1–43.0)	375

**Table 4:** Odds ratios (ORs) with 95% confidence intervals (CIs) for the prevalence of ESBL/AmpC-positive *E. coli* in broiler neck skin samples by production system. The conventional group (E) was used as the reference category (*OR* = 1).

Category	ESBL-AmpC/Total	OR (CI 95%)	*p*-Value
A	25/75 (33.3%)	0.50 (0.24, 1.00)	0.049
B	17/65 (26.2%)	0.36 (0.16, 0.76)	0.004
C	45/105 (42.9%)	0.75 (0.40, 1.40)	0.373
D	15/50 (30.0%)	0.43 (0.19, 0.96)	0.029
E	40/80 (50.0%)	1.00	-

**Table 5:** Distribution of detected ESBL and AmpC genes among 142 tested isolates.

Category	Detected Genes and Combinations	No. of Positive (%)	Total (%)
**ESBL genes**	*bla*_CTX-M-1_ * bla_CTX-M-1_ * + *bla*_TEM_ *bla*_CTX-M-9_ *bla*_CTX-M-9_ + *bla*_TEM_ *bla*_TEM_ *bla*_TEM_ + *bla*_SHV_ *bla*_CTX-M-1_ * + *bla*_TEM_ + *bla*_SHV_ *bla*_SHV_ *bla*_OXA_ *bla*_CTX-M-2_	35 (24.6) 23 (16.3) 3 (2.1) 9 (6.3) 2 (1.4) 20 (14.2) 1 (0.7) 35 (24.6) 0 (0.0) 0 (0.0)	128 (90.2)
**AmpC genes**	*bla*_CIT_ ^§^ *bla*_MOX_ *bla*_DHA_ *bla*_ACC_ *bla*_EBC_ *bla*_FOX_	11 (7.7) 0 (0.0) 0 (0.0) 0 (0.0) 0 (0.0) 0 (0.0)	11 (7.7)
**Negative**	Not detected	0 (0.00)	3 (2.1)
			142 (100.00)

* all *bla*_CTX-M-1_ are *bla*_CTM-M-15_-positive; ^§^ all *bla*_CIT_ are *bla*_CMY-2_-positive.

## Data Availability

The original contributions presented in this study are included in the article/Supplementary Material. Further inquiries can be directed to the corresponding author.

## Supplementary Materials

The following supporting information can be downloaded at: https://www.mdpi.com/article/10.3390/pathogens14121265/s1. Table S1. Distribution of minimum inhibitory concentrations (MICs) of *E. coli* isolates from MacConkey agar (n = 358) obtained using the EUVSEC panel. Percentages are shown in brackets. The gray-shaded areas indicate the range of concentrations tested for each antimicrobial. Black vertical bars indicate the epidemiological cut-off values (ECOFFs), according to the EFSA report (2024) [40].

## Abbreviations

The following abbreviations are used in this manuscript:

AMR	Antimicrobial Resistance
ATB-free	Antibiotic-free
CFU	Colony Forming Units
CI	Confidence Interval
CLSI	Clinical and Laboratory Standards Institute
COL	Colistin
*df*	Degrees of Freedom
DNA	Deoxyribonucleic Acid
*E. coli*	*Escherichia coli*
ECDC	European Centre for Disease Prevention and Control
ECOFF	Epidemiological Cut-Off Value
EFSA	European Food Safety Authority
EMA	European Medicines Agency
ETP	Ertapenem
EU	European Union
EUCAST	European Committee on Antimicrobial Susceptibility Testing
EUVSEC/EUVSEC2/EUVSEC3	Sensititre EU Surveillance *E. coli*/Salmonella panels
ESBL	Extended-Spectrum β-Lactamase
FEP	Cefepime
FOT	Cefotaxime
F/Cl	Cefotaxime/Clavulanic acid
FOX	Cefoxitin
GEN	Gentamicin
IMI	Imipenem
MEM	Meropenem
MIC	Minimum Inhibitory Concentration
MALDI-TOF MS	Matrix-Assisted Laser Desorption Ionization–Time of Flight Mass Spectrometry
NAL	Nalidixic Acid
NWT	Non-Wild Type
*OR*	Odds Ratio
PCR	Polymerase Chain Reaction
R	R statistical software
*SE*	Standard Error
SMX	Sulfamethoxazole
TBE	Tris–Borate–EDTA buffer
TET	Tetracycline
TGC	Tigecycline
TMP	Trimethoprim
TRM	Temocillin
TAZ	Ceftazidime
T/Cl	Ceftazidime/Clavulanic acid
TSA	Tryptic Soy Agar
WHO	World Health Organization
WT	Wild Type
*χ*^2^	Chi-squared statistic test

## Appendix A

**Table A1:** Selected resistance profiles of *E. coli* isolates collected on MacConkey agar from neck skin with CIs 95% in different production systems (A = Organic, B = ATB-free, C = ATB-free after conventional, D = ATB-free before conventional, E = Conventional). Groups A and B refer to slaughterhouse 1; groups C, D and E refers to slaughterhouse 2.

Antibiotic	A	B	C	D	E
**Ampicillin**	37.1%	41.7%	43.0%	68.8%	73.8%
	(26.8–48.9)	(30.1–54.3)	(33.7–52.8)	(54.7–80.1)	(63.2–82.1)
**Azithromycin**	0%	0%	2%	0%	7.5%
	(0.0–5.2)	(0.0–6.0)	(0.6–7.0)	(0.0–7.4)	(3.5–15.4)
**Cefotaxime**	8.6%	0%	3%	2.1%	8.8%
	(4.0–17.5)	(0.0–6.0)	(1.0–8.5)	(0.4–10.9)	(4.3–17.0)
**Ceftazidime**	7.1%	0%	2%	0%	10%
	(3.1–15.7)	(0.0–6.0)	(0.6–7.0)	(0.0–7.4)	(5.2–18.5)
**Ciprofloxacin**	28.6%	60%	50%	58.3%	67.5%
	(19.3–40.1)	(47.4–71.4)	(40.4–59.6)	(44.3–71.2)	(56.6–76.8)
**Chloramphenicol**	4.3%	13.3%	9%	16.7%	26.2%
	(1.5–11.9)	(6.9–24.2)	(4.8–16.2)	(8.7–29.6)	(17.9–36.8)
**Meropenem**	0%	0%	0%	0%	0%
	(0.0–5.2)	(0.0–6.0)	(0.0–3.7)	(0.0–7.4)	(0.0–4.6)
**Nalidixic acid**	28.6%	50%	46%	56.2%	62.5%
	(19.3–40.1)	(37.7–62.3)	(36.6–55.7)	(42.3–69.3)	(51.5–72.3)
**Trimethoprim**	21.4%	30%	30%	47.9%	43.8%
	(13.4–32.4)	(19.9–42.5)	(21.9–39.6)	(34.5–61.7)	(33.4–54.7)
**Tigecycline**	8.6%	1.7%	2%	6.2%	1.2%
	(4.0–17.5)	(0.3–8.9)	(0.6–7.0)	(2.1–16.8)	(0.2–6.7)
**Tetracycline**	40%	36.7%	49%	54.2%	47.5%
	(29.3–51.7)	(25.6–49.3)	(39.4–58.7)	(40.3–67.4)	(36.9–58.3)
**Colistin**	0%	0%	1%	0%	1.2%
	(0.0–5.2)	(0.0–6.0)	(0.2–5.4)	(0.0–7.4)	(0.2–6.7)
**Gentamicin**	0%	3.3%	3%	2.1%	6.2%
	(0.0–5.2)	(0.9–11.4)	(1.0–8.5)	(0.4–10.9)	(2.7–13.8)
**Sulfamethoxazole**	42.9%	45%	54%	66.7%	72.5%
	(31.9–54.5)	(33.1–57.5)	(44.3–63.4)	(52.5–78.3)	(61.9–81.1)
